# Network pharmacology and molecular docking analysis on Shenfu Qiangxin indicate mTOR is a potential target to treat heart failure

**DOI:** 10.1186/s40001-024-01732-8

**Published:** 2024-03-13

**Authors:** Peilin Zou, Jiajun Li, Yucong Zhang, Zonghao Qian, Hao Nie, Ni Yang, Le Zhang, Li Lin, Dewei Peng, Cuntai Zhang

**Affiliations:** 1 Department of Geriatrics, Tongji Hospital, Tongji Medical College, Huazhong University of Science and Technology, Wuhan, 430030 China; 2 The Second Clinical School, Tongji Hospital, Tongji Medical College, Huazhong University of Science and Technology, Wuhan, China; 3 Division of Cardiothoracic and Vascular Surgery, Tongji Hospital, Tongji Medical College, Huazhong University of Science and Technology, Wuhan, Hubei China; 4 Division of Cardiology, Department of Internal Medicine, Tongji Hospital, Tongji Medical College, Huazhong University of Science and Technology, Wuhan, China

**Keywords:** Shenfu Qiangxin, Heart failure, mTOR, Molecular docking, Network pharmacology

## Abstract

**Background:**

Heart failure (HF) is one of the major causes of mortality worldwide with high recurrence rate and poor prognosis. Our study aimed to investigate potential mechanisms and drug targets of Shenfu Qiangxin (SFQX), a cardiotonic-diuretic traditional Chinese medicine, in treating HF.

**Methods:**

An HF-related and SFQX-targeted gene set was established using disease-gene databases and the Traditional Chinese Medicine Systems Pharmacology database. We performed gene function and pathway enrichment analysis and constructed protein–protein interaction (PPI) network to investigate the potential mechanisms. We also performed molecular docking to analyze the interaction patterns between the active compounds and targeted protein.

**Results:**

A gene set with 217 genes was identified. The gene function enrichment indicated that SFQX can regulate apoptotic process, inflammatory response, response to oxidative stress and cellular response to hypoxia. The pathway enrichment indicated that most genes were involved in PI3K–Akt pathway. Eighteen hub target genes were identified in PPI network and subnetworks. mTOR was the key gene among hub genes, which are involved in PI3K–Akt pathway. The molecular docking analysis indicated that 6 active compounds of SFQX can bind to the kinase domain of mTOR, which exerted potential therapeutic mechanisms of SFQX in treating HF.

**Conclusions:**

The results of network pharmacology analysis highlight the intervention on PI3K–Akt pathway of SFQX in the treatment of HF. mTOR is a key drug target to help protect myocardium.

**Supplementary Information:**

The online version contains supplementary material available at 10.1186/s40001-024-01732-8.

## Background

Heart failure (HF) is a series of chronic syndromes in which the heart muscle doesn't pump enough blood because of decreased function and (or) abnormal heart structure [1]. The main symptoms include fatigue, fluid retention and dyspnea. It is a serious condition, which needs active and effective intervention to prevent fatal complications [2]. For decades, many drugs have been used in treating chronic HF (CHF). Although some drugs are effective to improve the symptoms, the compliance is still poor due to long-term use and adverse effects [3]. Unfortunately, the incidence of CHF keeps increasing with the aging global population [4]. Therefore, finding a pharmacotherapy or nonpharmacological treatment with satisfactory safety and efficacy for treating CHF is a hot topic, which receives wide attention worldwide.

Traditional Chinese medicine (TCM) has been used in HF treatment for ages [5]. It is an idea HF treatment, because it is multilevel multitargeted with few side effects [6]. Shenfu Qiangxin (SFQX) capsule is a cardiotonic-diuretic medicine approved by the China Food and Drug Administration and recommended by expert consensus for the treatment of HF [7]. SFQX is composed of six Chinese herbal extracts: Ginseng (Renshen, RS), Aconiti Lateralis Radix Praeparata (Fuzi, FZ), Mori Cortex (Sangbaipi, SBP), Polyporus Umbellatus (Zhuling, ZL), Descurainiae Semen (Tinglizi, TLZ) and Radix Rhei Et Rhizome (Dahuang, DH). It is reported that SFQX may alleviate oxidative stress-induced myocardial injury by regulating SIRT4/FOXO3a signaling in animal and cell studies [8]. However, the detail mechanisms of SFQX in treating HF remain unclear.

Network pharmacology can construct and visualize ‘multi-gene-target-pathway’ interaction network to assess the molecular mechanism of agents by integrating medicine and computer science [9], especially for the assessment of TCM with complicated matrix nature [10, 11]. In this study, a comprehensive network pharmacology-based analysis was used to demonstrate the mechanisms of SFQX in treating HF. We also identified the active components and the key targets of SFQX in treating HF.

Mechanistic Target of Rapamycin (mTOR) is involved in the regulation of cell growth, cell metabolism and nutrient sensing. Many age-related pathologies are partly caused by dysregulation of mTOR signaling, such as cardiac dysfunction and HF [12]. Molecular docking is the process that a small ligand spatially docks into a macromolecular, such as protein. It can be used for structure-based drug design scoring the complementary values of binding sites [13]. In the current study, we also investigated the potential mechanisms of SFQX in HF using molecular docking. mTOR was found to be the hub gene in SFQX-target genes, which suggested a new target for HF treatment by SFQX.

## Materials and methods

### Obtaining the SFQX target and HF-related gene set

The main ingredients of SFQX were obtained from the Traditional Chinese Medicine Systems Pharmacology (TCMSP) database (https://www.tcmsp-e.com/) [14] by searching the “Herb name”. Active compounds were then filtered by setting the oral bioavailability (OB) > 40% and the drug-like (DL) index > 0.30. The three-dimensional structure of each active compound was obtained from PubChem [15]. The compounds without available three-dimensional molecular structures were excluded. SwissTargetPrediction was then used to predict potential targets according to the three-dimensional structure of each compound [16]. Target genes with probability greater than 0.10 were considered as potential target genes of each compound. The compounds without such target genes were also excluded.

HF-related genes were searched in five databases: Genecards database (https://www.genecards.org/) [17], OMIM database (https://omim.org/) [18], TTD database (http://db.idrblab.net/ttd/) [19], DrugBank database (https://www.drugbank.ca/) [20], and DisGeNet database (https://www.disgenet.org/home/) [21]. Genes with Gifts > 40 and Relevance score > 10 were filtered from Genecards database. The HF-related gene set was established by combining all the search results.

The HF-related and SFQX-target gene set was generated by intersecting the HF-related gene set and the SFQX-target gene set.

### Compound-target pharmacology network and enrichment analysis

Using Cytoscape version 3.8.0, a target-compound network was constructed based on the SFQX-HF target gene set and the SFQX compound set [22]. Gene ontology (GO) and Kyoto Encyclopedia of Genes and Genomes (KEGG) pathway enrichment analysis were performed using KOBAS 3.0 (https://kobas.cbi.pku.edu.cn) to investigate the potential mechanisms and key signaling pathways [23]. The genes in mostly enriched pathway, which was also believed to be involved in HF, were further analyzed.

### Protein–protein interaction (PPI) network and core subnetwork

The PPI network was constructed using STRING database [24]. The parameter was set as moderate confidence (0.400). The PPI network was downloaded from STRING database and subsequently imported into Cytoscape to identify the core subnetwork using CytoNca plugin [25] and CytoHubba plugin [26]. In detail, according to the score file calculated by CytoNca plugin, genes with each score of Betweenness, Closeness, Degree, Eigenvector, LAC, network scores higher than the median value were filtered for the construction of subnetwork. This subnetwork was then calculated by using CytoHubba plugin to further rank the key gene. Combining the analysis results by CytoNca plugin and CytoHubba plugin, a key target gene was identified.

### Molecular docking

The most core gene from the above analysis was then selected for molecular docking. The crystal structure of the receptor protein that is coded by this gene was downloaded at Protein Data Bank (https://www.rcsb.org/). The structure of molecule ligands was obtained from Scifinder Scholar. Discovery Studio 2016 was used to carry out hydrogenation of protein. And AutoDockTools-1.5.6 was used to charge calculation and determine parameters of the protein docking area. Then, the minimizing energy of molecule ligands was calculated and exported by ChemBio3D 19.0 and AutoDockTools. Finally, Molecular docking of ligands and receptor protein were performed by Autodock Vina [27]. And the docking results were shown in Discovery Studio.

## Results

### Screening of active compounds and target genes

**Fig. 1:**
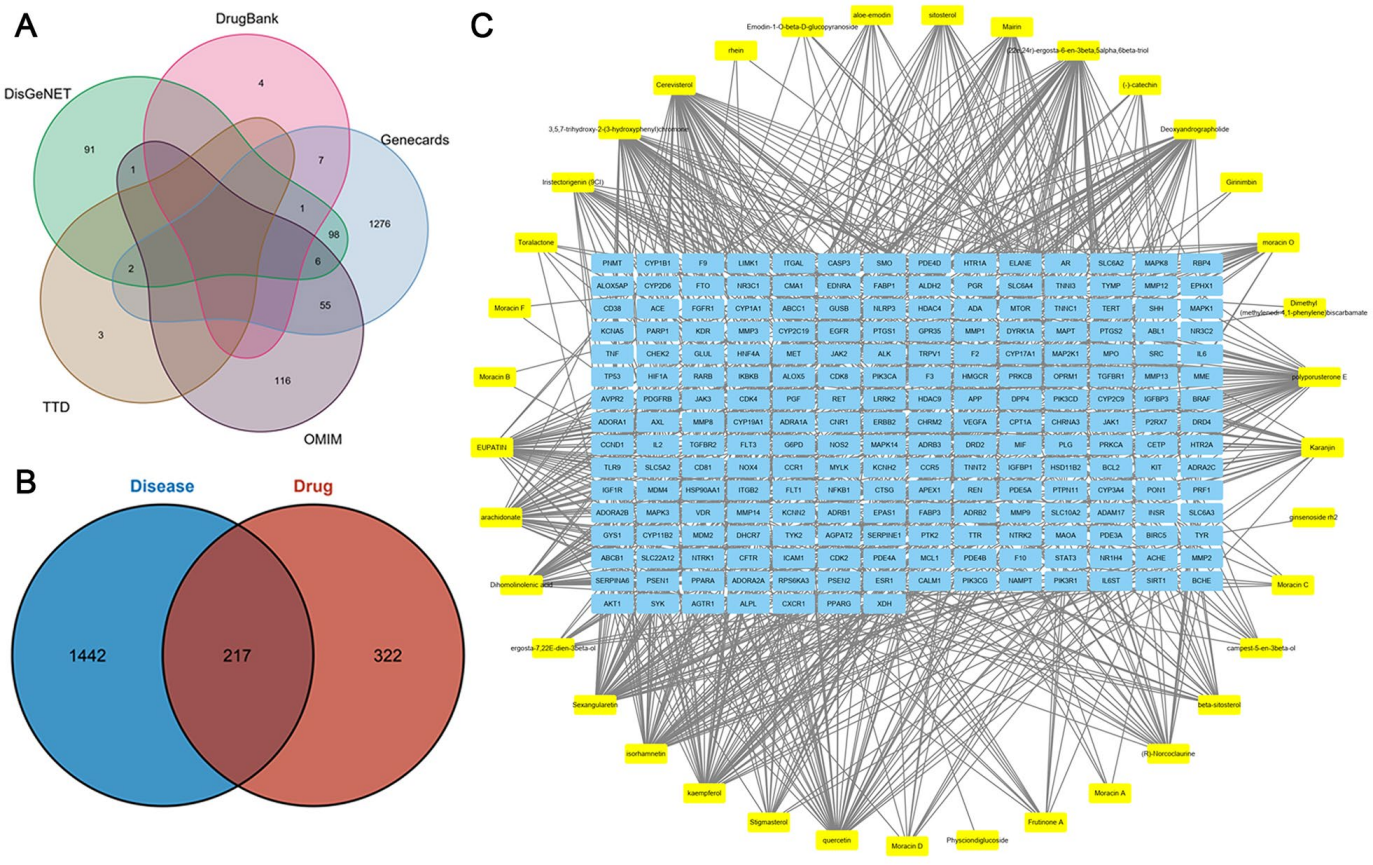
Identification of the drug-target interaction. **A** Identification of the HF-related genes by taking a union of all the results from 5 databases; **B** identification of the drug-target disease-related genes by taking an intersection of SFQX target genes and HF-related genes; **C** the compound-targets interaction pharmacology network of SFQX and interaction genes. Circle represents the molecule active compounds in SFQX. Each yellow rectangle represents a traditional Chinese medicine compound. Each blue rectangle represents a HF-related target gene. Edges represent the interaction between the molecule compounds and the target genes. *HF* heart failure, *SFQX* Shenfu Qiangxin

By using the TCMSP database and SwissTargetPrediction, 39 compounds with 539 target genes were identified (Additional file 1: Fig. S1). Besides, 1445, 178, 5, 13 and 199 HF-related genes were obtained from Genecards, OMIM, TTD, DrugBank and DisGeNet database, respectively. After we removed duplication and combined the search results, a gene set with 1659 HF-related genes was constructed (Fig. 1A). And we finally acquired the SFQX target and HF-related gene set with 217 genes included by taking an intersection of the SFQX-target genes and HF-related genes (Fig. 1B). The 217 intersection genes were target genes of 37 compounds (Additional file 1: Table S1). The target-compound network with 254 nodes and 822 edges is visualized in Fig. 1C. One gene was targeted by several active compounds while one compound could also target multiple gene. Among 217 genes, CYP19A1 and ESR1 were the most targeted gene by SFQX compounds. Both of them were targeted by 21 compounds.

### Enrichment analysis

**Fig. 2:**
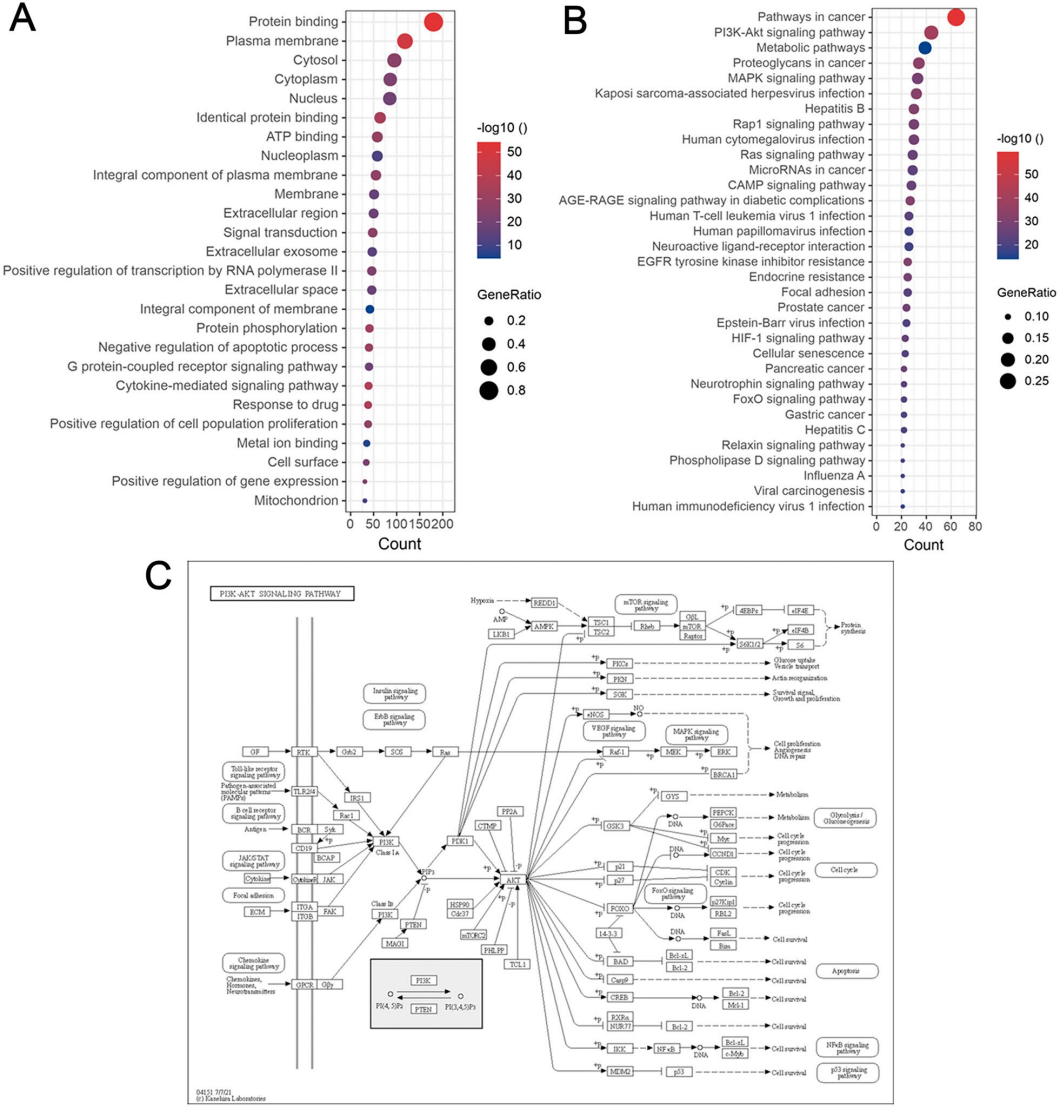
Enrichment analysis and pathway map. **A** GO enrichment analysis of the target genes. Gene ratio refers to the ratio of enriched genes to all target genes, and counts refer to the number of the enriched genes; **B** KEGG enrichment analysis of the target genes. Gene ratio refers to the ratio of enriched genes to all target genes. Counts refer to the number of the enriched genes; **C** pathway map of PI3K–Akt signaling pathway. *GO* gene ontology, *KEGG* Kyoto Encyclopedia of Genes and Genomes

The underlying gene ontology of the 217 target genes was discovered by GO enrichment analysis. 1546 significant GO terms with corrected *P*-value < 0.05 were identified. The terms with enrichment gene count > 30 are shown in Fig. 2A. The GO terms indicated that these target genes were involved in protein binding and plasma membrane construction. In addition, some GO terms, such as negative regulation of apoptotic process, inflammatory response, response to oxidative stress and cellular response to hypoxia, were associated with the development of HF, which indicated that these target genes may be involved in the regulation of HF.

The pathways, which the 217 target genes were enriched in, were discovered by KEGG enrichment analysis. 212 KEGG pathways with corrected *P*-value < 0.05 were significantly enriched. Except for the pathways in cancer, the most enriched pathway was PI3K–Akt signaling pathway, which is reported to play an important role in HF [28]. The 44 genes enriched in PI3K–Akt signaling pathway are shown in Additional file 1: Table S2. The bubble plot of the KEGG pathways with enrichment gene count > 20 is shown in Fig. 2B and the map of the PI3K–Akt signaling pathway is shown in Fig. 2C.

### PPI network and core subnetwork

**Fig. 3:**
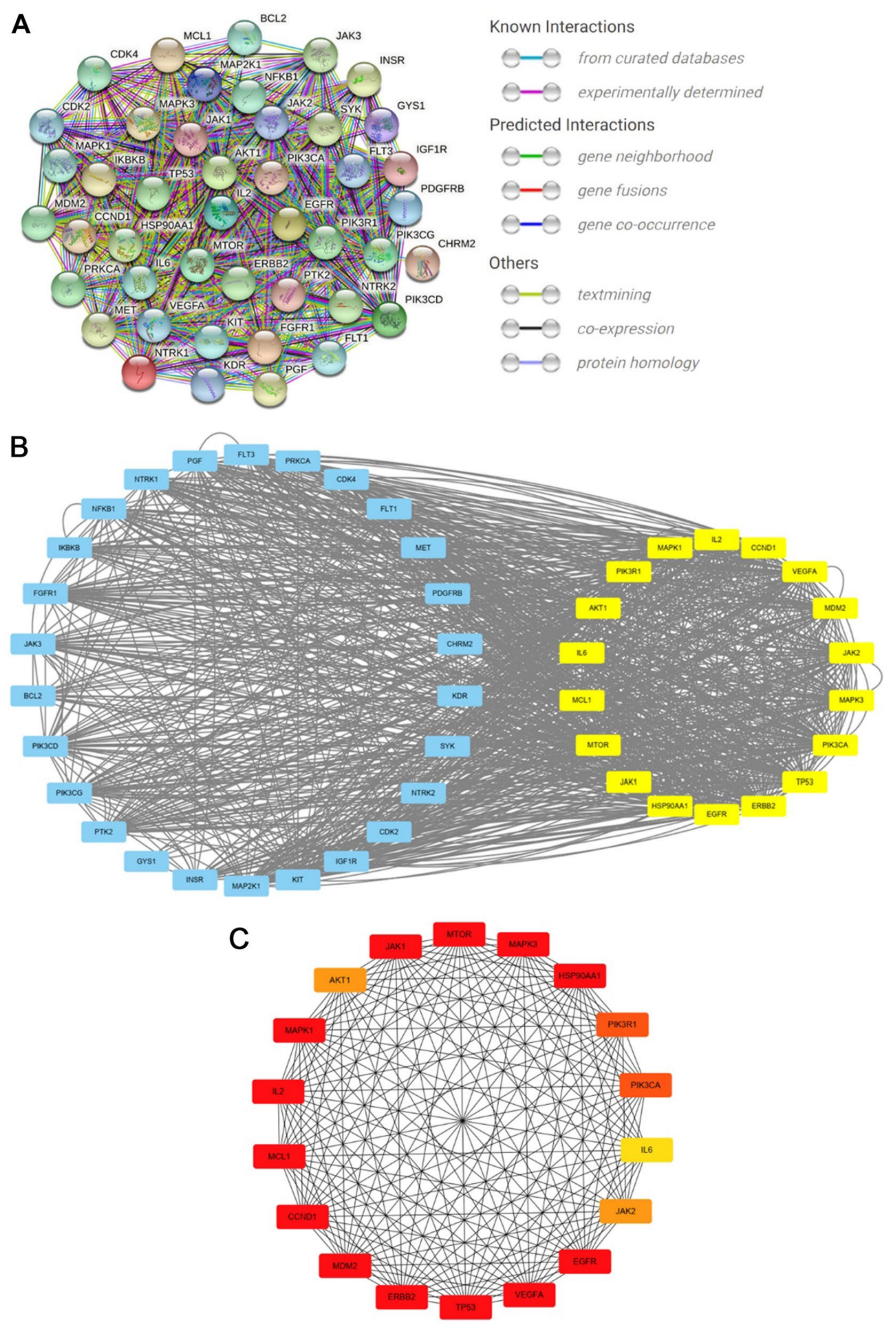
PPI network and identification of key subnetwork. **A** PPI network for the proteins encoded by target genes enriched in PI3K–Akt signaling pathway exported from STRING database; **B** a subnetwork constructed by filtration via CytoNca. The yellow nodes were screened with a score higher than the median. **C** Rank of genes by CytoHubba. The darker red colour refers to higher rank. *PPI* protein–protein interaction

PPI network from STRING database for proteins encoded by 217 genes is shown in Additional file 1: Fig. S2. PPI network for the proteins encoded by target genes enriched in PI3K–Akt signaling pathway had complex interactions (Fig. 3A). This PPI network was imported into Cytoscape for the identification of core subnetwork. A core subnetwork composed of 18 genes were identified using CytoNca (Fig. 3B, Additional file 1: Table S3). These 18 target genes were further ranked by CytoHubba (Fig. 3C, Additional file 1: Table S4). After combining the analysis results by CytoNca plugin and CytoHubba plugin, MTOR was identified as the key target gene. It ranked first in CytoNca and second in CytoHubba.

### Molecular docking of active compounds and MTOR encoding protein

**Fig. 4:**
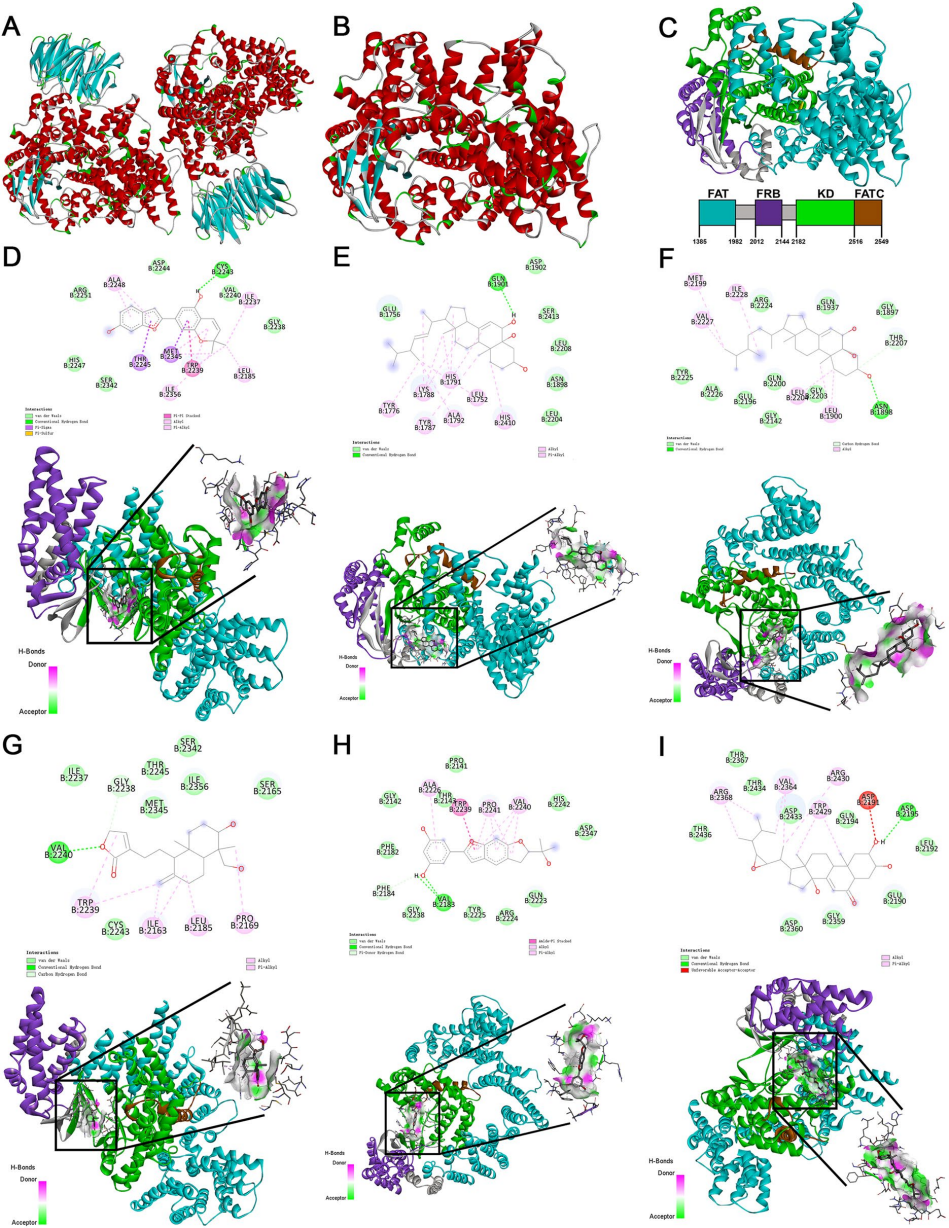
Molecular docking. **A** Original crystal structure of the mTOR downloaded from Protein Data Bank (4JSV); **B** the monomer structure of mTOR; **C** the structure of mTOR with each domain marked by different colour; molecular docking for mTOR with moracin D (**D**), cerevisterol (**E**), (22e,24r)-ergosta-6-en-3beta,5alpha,6beta-triol (**F**), deoxyandrographolide (**G**), moracin O (**H**) and polyporusterone E (**I**), on the top shows the surface of the receptor protein and 3D structure of the ligand, at the bottom shows the detail binding bond of each ligand with receptor protein

The crystal structure of the mTOR was downloaded at Protein Data Bank (4JSV). The original structure of 4JSV is a homodimer, which contains two identical complexes of atypical kinase mTOR and ligand mLST8 (Fig. 4A). We removed one of the complexes and the ligand mLST8 to obtain the monomer structure as the receptor protein encoded by MTOR for further molecular docking (Fig. 4B, C). In the compound-target interaction network, six active compounds targeted mTOR protein, including moracin D (from Sangbaipi), cerevisterol (from Zhuling), (22e,24r)-ergosta-6-en-3beta,5alpha,6beta-triol (from Zhuling), deoxyandrographolide (from Fuzi), moracin O (from Sangbaipi) and polyporusterone E (from Zhuling) (Additional file 1: Fig. S3). Using Autodock Vina, several binding sites in mTOR for each compound were predicted. The docking results indicated that all these 6 compounds could easily bind to the protein kinase domain of mTOR (Fig. 4D–I) through several bonds. The molecular docking binding energy for these sites is recorded in Table 1.

**Table 1:** Molecular docking binding energy of each compound with mTOR

Compound name	Molecular docking binding energy
Moracin D	− 8.3
Cerevisterol	− 7.5
(22e,24r)-Ergosta-6-en-3beta,5alpha,6beta-triol	− 7.2
Deoxyandrographolide	− 7.0
Moracin O	− 7.5
Polyporusterone E	− 8.7

## Discussion

HF is the end stage of cardiac diseases, such as cardiomyopathy, high blood pressure, coronary heart disease, and acute myocardial infraction [29]. A failing heart can’t pump sufficiently and provide enough blood supply [30]. The conventional HF management agents are diuretics, β-adrenergic blockers, and angiotensin-converting enzyme inhibitors [31]. Unfortunately, severe side effects may occur during the long-time use of these chemical agents, such as hypotension, fluid depletion and electrolyte depletion [32]. Therefore, TCM can be used as alternative agents in treating HF with fewer side effects and lower cost [33].

Several TCMs have been used in the management of HF with satisfactory effect [5], for example, Zhenwu tang, Shengmai san, Baoyuan tang, Xuefuzhuyu tang, Tinglidazaoxiefei tang, Danshen yin, Taohongsiwu tang. Meanwhile, several Chinese patent drugs have been successfully produced by standardized procedures and are widely used in health care industry, such as Qishenyiqi dripping pill, Fufang danshen dripping pill, Danqi pill, Qili qiangxin capsule, Shengmai capsule [34, 35]. Although TCMs are commonly used as complementary therapy to treat HF, there is currently evidence to support the use of TCM alone in treating HF. The LVEF of HF patients treated with Xinmailong for 15 days was increased from 36.9 to 46.4% [36], which indicated that TCM can be used as an independent treatment for HF.

In clinical study, SFQX combined with recombinant human brain natriuretic peptide can improve the cardiac function, and decrease myocardial enzyme indexes and myocardial damage markers of HF patients [37]. Moreover, it is reported that SFQX can protect heart by correcting electrolyte disturbances, reducing sodium and water retention, and inhibiting apoptosis and autophagy of myocardial cells in several animal experiments [38]. In this study, an SFQX target HF-related gene set with 217 target genes included was constructed by analyzing the active components of SFQX. GO analysis revealed that SFQX can regulate the process which are involved in the development of HF, such as negative regulation of apoptotic process [39], inflammatory response [40], response to oxidative stress [41] and cellular response to hypoxia [42]. KEGG analysis identified several signal pathways associated with HF, in which PI3K–Akt pathway is the pathway with the largest number of genes enriched in. PPI network and critical subnetwork analyses found 18 hub target genes out of 44 genes, which were involved in PI3K–Akt pathway. Among all the hub target genes, MTOR was the most significant gene. And we performed molecular docking to analysis the interaction between mTOR and active compounds in SFQX. The results demonstrated the potential roles of SFQX in treating HF by bioinformatics analysis, and provided an overview on the mechanism of SFQX, which may help targeted drug design and basic research of HF treatment.

We identified several active compounds of SFQX from TCMSP database. Renshen and Fuzi are important ingredients in SFQX. The major active components in ginseng are ginsenosides, which have been shown to inhibit HF in several experimental models of both left and right ventricular hypertrophy or failure [43]. For NYHA Class II to IV HF patients, the administration of a water extract of *P. ginseng* combined with standard HF therapy for 14 days results in the improvement in several parameters, including quality of life scores, which is determined by a questionnaire, and left ventricular function. It can also reduce plasma cytokine levels, and indices of hepatic injury [44]. A systematic review and meta-analysis on the efficacy and safety of Fuzi Formulae, a prescription containing Fuzi as major ingredient, in treating HF analyzes 12 high-quality randomized clinical trials with 1490 participants, in which the control group received standardized treatment with or without placebo, while the intervention group received standardized treatment with Fuzi Formulae. The results indicate statistical benefits from Fuzi Formulae in reducing plasma NT-proBNP level and improving the efficacy on NYHAfc and LVEF. Moreover, the patients’ prognosis and life quality are also improved and patients’ risks in readmission and death for HF are reduced [45].

In current study, mTOR was identified as the key SFQX target and HF-related protein, which involves in PI3K–Akt signaling pathway. Accumulated studies have proven that phosphoinositol-3 kinase (PI3K)/Akt signaling pathway is involved in regulating the occurrence, progression and pathological formation of cardiac fibrosis via regulating cell survival, apoptosis, growth, cardiac contractility and even the transcription of related genes through a series of molecules including mammalian target of rapamycin (mTOR), glycogen synthase kinase 3 (GSK-3), forkhead box proteins O1/3 (FoxO1/3), and nitric oxide synthase (NOS) [28]. Six compounds from SFQX could easily bind to the protein kinase domain of mTOR. The mTOR mainly belongs to PI3K-related kinases with conserved domain [46]. The mTOR protein consists of several domains including HEAT repeats, a FAT domain, a protein kinase domain, an FRB domain and a FATC domain [47, 48]. The kinase domain is essential for mTOR function [49]. In normal cells, mTOR is stimulated by amino acids, stress, redox sensors, oxygen, growth factors, or energy. The active mTOR can promote cellular anabolism to synthetize several macromolecules, including lipids, proteins and nucleic acids in response to those environmental stimuli. The mTOR can regulate metabolic pathways by integrating these anabolic processes in cell metabolism, growth, proliferation, and autophagy [50]. The mTOR signaling plays an important role in aging. The dysregulation of mTOR is associated with many age-related diseases, such as cardiac dysfunction and HF [12]. MTOR is reported to regulate the upstream signals of autophagy, significantly improved the cardiac function with HF by inhibiting apoptosis and activating autophagy [51]. mTOR complex 1 (mTORC1) is involved in the functional and structural deterioration of heart [52, 53]. The inhibition of mTORC1-related pathway by rapamycin [50, 54] or caloric restriction [55] can rejuvenate the senescent heart or ameliorate cardiovascular function and inhibit cardiac aging pathologies, such as cardiac fibrosis and inflammation. Our current study demonstrated that some compounds from SFQX can bind to the protein kinase domain of mTOR, which indicated that SFQX may help the intervention of cardiac aging and heart failure. In clinical settings, SFQX (5.4 g) was given twice or three times a day, combined with standardized chemical medicine treatment, in treating HF patients [56].

In this study, we analyzed the potential therapeutic mechanisms of the SFQX in treating HF. The results emphasize the intervention on PI3K–Akt pathway by SFQX in the treatment of HF. However, there was a lack of experimental validation of our results, which was the main limitation of our study. Future clinical study should assess the efficacy and safety of SFQX in treating HF, either used alone or combined with standard medical treatment. Moreover, the detailed mechanism of the compounds in SFQX still needs further investigation, which could help the design of anti-HF drugs.

## Conclusions

We investigated the potential mechanisms of SFQX by performing pharmacology network and molecular docking analyses. PI3K–Akt pathway, especially mTOR-related signaling pathway, is involved in the mechanism of SFQX in treating HF.

### Supplementary Information

**Additional file 1: Figure S1.** The compound‐targets interaction pharmacology network of Shenfu Qiangxin. Circle represents the molecule active compounds in Shenfu Qiangxin. Each yellow rectangle represents a traditional Chinese medicine compound. Each blue rectangle represents a target gene. **Figure S2.** Protein‐protein interaction network derived from STRING database for proteins encoded by 217 intersection genes. **Figure S3.** Two-dimensional structure of compounds used for molecular docking with mTOR. A. moracin D; B. cerevisterol; C. (22e,24r)-ergosta-6-en-3beta,5alpha,6beta-triol; D. deoxyandrographolide; E. moracin O; F. polyporusterone E. **Table S1.** Chemical information for Shenfu Qiangxin compounds related to heart failure. **Table S2.** Target genes enriched in PI3K–Akt signaling pathway. **Table S3.** The core-subnetwork analysis of genes enriched in PI3K–Akt signaling pathway by CytoNca. **Table S4.** Ranking results of 18 target genes by CytoHubba.

## Data Availability

All research data are included in the paper, with the absence of shared data.

## Abbreviations

HF: Heart failure
SFQX: Shenfu Qiangxin
GO: Gene ontology
KEGG: Kyoto Encyclopedia of Genes and Genomes
PPI: Protein–protein interaction
CHF: Chronic heart failure
TCM: Traditional Chinese medicine
mTOR: Mechanistic Target of Rapamycin
TCMSP: Traditional Chinese Medicine Systems Pharmacology
DL: Drug like
OB: Oral bioavailability
AMI: Acute myocardial infraction
PIKK: PI3K-related kinases
mTORC1: MTOR complex 1
