# Supplementary material for: Network pharmacology and molecular docking analysis on Shenfu Qiangxin indicate mTOR is a potential target to treat heart failure
Source: Eur J Med Res. 2024 Mar 13;29:173. doi: 10.1186/s40001-024-01732-8 (PMC10935835; doi:10.1186/s40001-024-01732-8)
Supplement: Supplementary file 1 — Additional file 1: Figure S1. The compound‐targets interaction pharmacology network of Shenfu Qiangxin. Circle represents the molecule active compounds in Shenfu Qiangxin. Each yellow rectangle represents a traditional Chinese medicine compound. Each blue rectangle represents a target gene. Figure S2. Protein‐protein interaction network derived from STRING database for proteins encoded by 217 intersection genes. Figure S3. Two-dimensional structure of compounds used for molecular docking with mTOR. A. moracin D; B. cerevisterol; C. (22e,24r)-ergosta-6-en-3beta,5alpha,6beta-triol; D. deoxyandrographolide; E. moracin O; F. polyporusterone E. Table S1. Chemical information for Shenfu Qiangxin compounds related to heart failure. Table S2. Target genes enriched in PI3K–Akt signaling pathway. Table S3. The core-subnetwork analysis of genes enriched in PI3K–Akt signaling pathway by CytoNca. Table S4. Ranking results of 18 target genes by CytoHubba. [file 40001_2024_1732_MOESM1_ESM.docx]

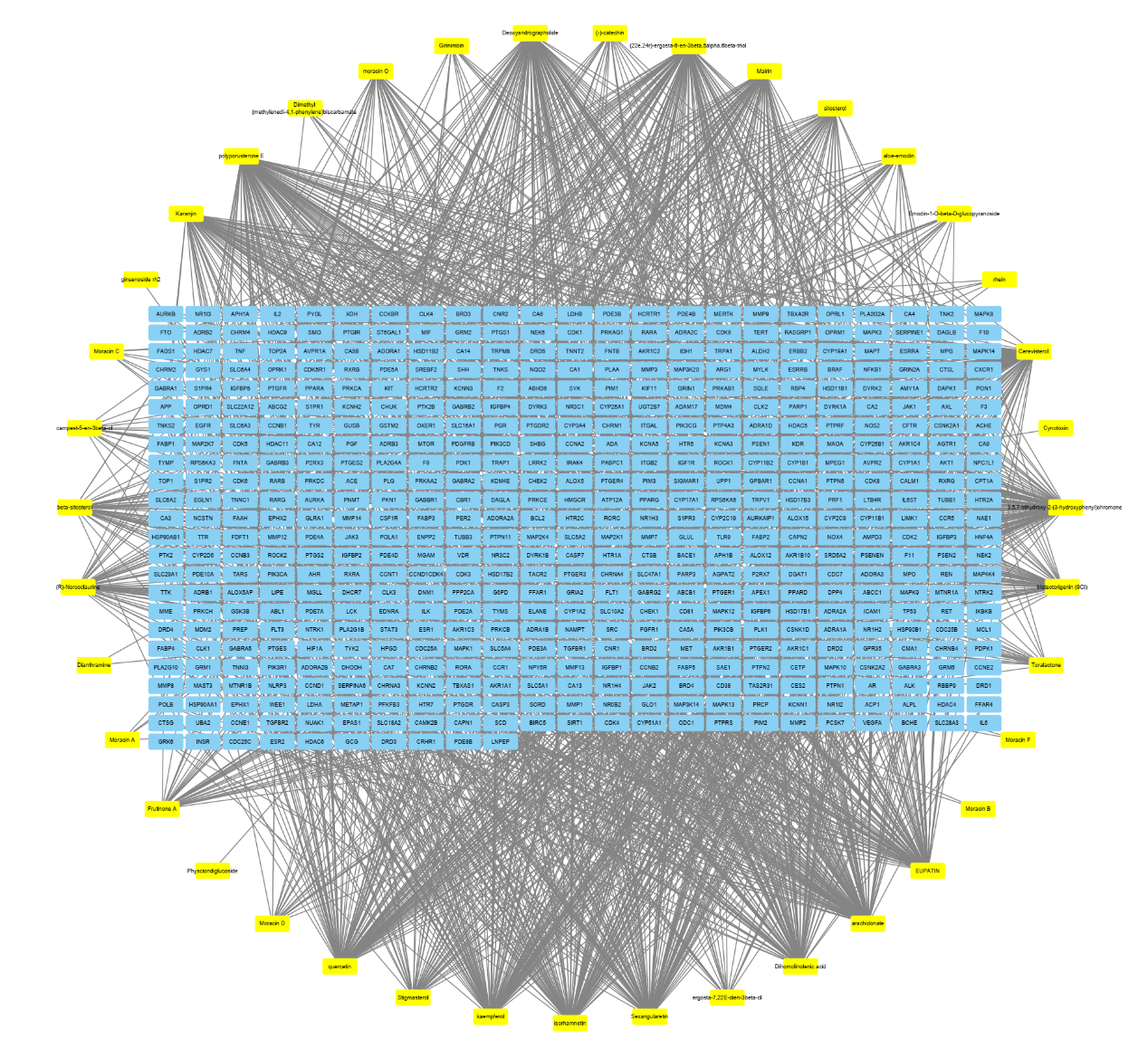


Figure S1 The compound‐targets interaction pharmacology network of Shenfu Qiangxin. Circle represents the molecule active compounds in Shenfu Qiangxin. Each yellow rectangle represents a traditional Chinese medicine compound. Each blue rectangle represents a target gene.


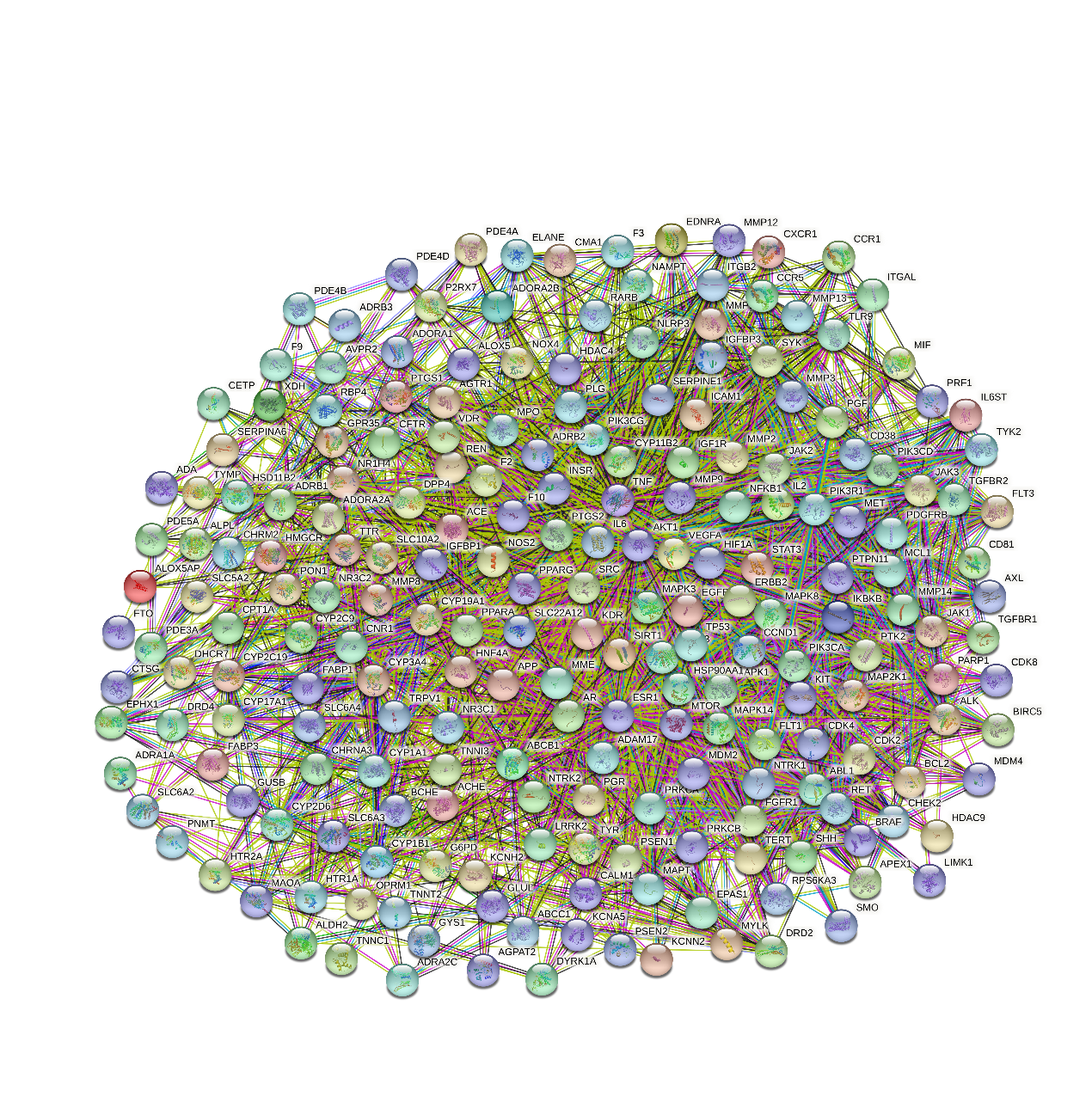


Figure S2 Protein‐protein interaction network derived from STRING database for proteins encoded by 217 intersection genes


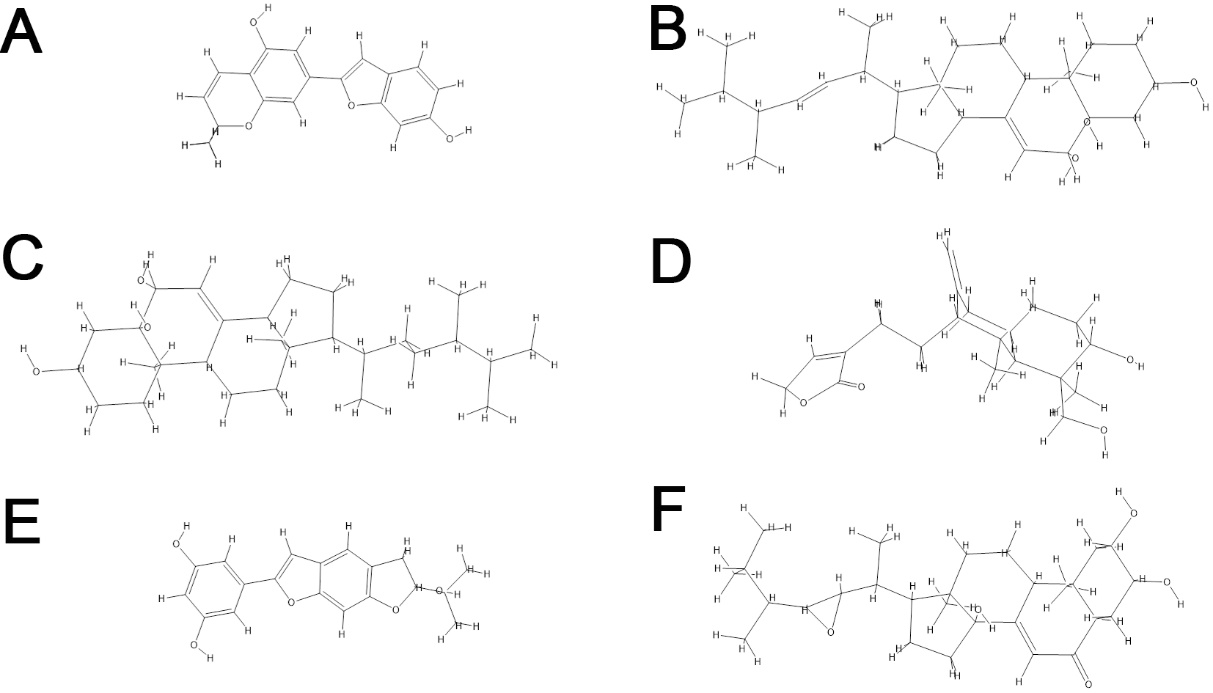


Figure S3 Two-dimensional structure of compounds used for molecular docking with mTOR. A. moracin D; B. cerevisterol; C. (22e,24r)-ergosta-6-en-3beta,5alpha,6beta-triol; D. deoxyandrographolide; E. moracin O; F. polyporusterone E.

Table S1 Chemical information for Shenfu Qiangxin compounds related to heart failure

| Pubchem ID* | Mol ID** | Molecule name | OB | DL |
| --- | --- | --- | --- | --- |
| 10168 | MOL002268 | rhein | 47.06521 | 0.27678 |
| 10207 | MOL000471 | aloe-emodin | 83.37964 | 0.2409 |
| 64971 | MOL000211 | Mairin | 55.37707 | 0.7761 |
| 73160 | MOL000096 | (-)-catechin | 49.67639 | 0.24162 |
| 96943 | MOL005356 | Girinimbin | 61.2153 | 0.31484 |
| 99337 | MOL012681 | Dimethyl (methylenedi-4,1-phenylene)biscarbamate | 50.84052 | 0.2625 |
| 100633 | MOL002398 | Karanjin | 69.55687 | 0.33616 |
| 119307 | MOL005344 | ginsenoside rh2 | 36.31951 | 0.55868 |
| 155248 | MOL003857 | Moracin C | 82.13155 | 0.28665 |
| 173183 | MOL005043 | campest-5-en-3beta-ol | 37.57682 | 0.71481 |
| 222284 | MOL000358 | beta-sitosterol | 36.91391 | 0.75123 |
| 440988 | MOL002419 | (R)-Norcoclaurine | 82.54295 | 0.20872 |
| 441839 | MOL012714 | Moracin A | 64.38875 | 0.2297 |
| 441965 | MOL005321 | Frutinone A | 65.90373 | 0.34184 |
| 442762 | MOL002259 | Physciondiglucoside | 41.64856 | 0.63145 |
| 641378 | MOL003858 | Moracin D | 60.92843 | 0.38454 |
| 5280343 | MOL000098 | quercetin | 46.43335 | 0.27525 |
| 5280794 | MOL000449 | Stigmasterol | 43.82985 | 0.75665 |
| 5280863 | MOL000422 | kaempferol | 41.88225 | 0.24066 |
| 5281654 | MOL000354 | isorhamnetin | 49.60438 | 0.306 |
| 5281698 | MOL002514 | Sexangularetin | 62.85792 | 0.2968 |
| 5283628 | MOL000282 | ergosta-7,22E-dien-3beta-ol | 43.50709 | 0.71939 |
| 5312529 | MOL003927 | Dihomolinolenic acid | 44.1103 | 0.20398 |
| 5312542 | MOL005320 | arachidonate | 45.57325 | 0.20491 |
| 5317287 | MOL002235 | EUPATIN | 50.80308 | 0.40804 |
| 5319887 | MOL003856 | Moracin B | 55.85088 | 0.23225 |
| 5319889 | MOL003860 | Moracin F | 53.81211 | 0.23084 |
| 5321980 | MOL002281 | Toralactone | 46.46436 | 0.23965 |
| 5488781 | MOL003758 | Iristectorigenin (9CI) | 71.54841 | 0.33954 |
| 9860659 | MOL012800 | 3,5,7-trihydroxy-2-(3-hydroxyphenyl)chromone | 59.71453 | 0.23961 |
| 10181133 | MOL000279 | Cerevisterol | 37.96383 | 0.77061 |
| 11968447 | MOL002288 | Emodin-1-O-beta-D-glucopyranoside | 44.80639 | 0.79742 |
| 12303645 | MOL000359 | sitosterol | 36.91391 | 0.7512 |
| 14236575 | MOL000796 | (22e,24r)-ergosta-6-en-3beta,5alpha,6beta-triol | 30.19604 | 0.76433 |
| 15922991 | MOL002395 | Deoxyandrographolide | 56.3041 | 0.31451 |
| 42603436 | MOL012719 | moracin O | 62.32611 | 0.44467 |
| 44575602 | MOL000820 | polyporusterone E | 45.71303 | 0.85389 |

* ID for compounds indexed in Pubchem database.

** ID for compound indexed in Traditional Chinese Medicine Systems Pharmacology database.

OB, oral bioavailability; DL, drug‐like index.

Table S2 Target genes enriched in PI3K-Akt signaling pathway

| Common name | | | | | | |
| --- | --- | --- | --- | --- | --- | --- |
| PDGFRB | PTK2 | IL2 | EGFR | IKBKB | AKT1 | INSR |
| MCL1 | FLT3 | FLT1 | PGF | PIK3CA | PIK3CD | NTRK1 |
| NTRK2 | PIK3CG | CCND1 | MAPK3 | MAPK1 | JAK2 | JAK3 |
| PIK3R1 | JAK1 | MTOR | NFKB1 | KDR | ERBB2 | IGF1R |
| PRKCA | CHRM2 | GYS1 | VEGFA | MDM2 | FGFR1 | TP53 |
| SYK | MAP2K1 | KIT | IL6 | MET | CDK2 | CDK4 |
| HSP90AA1 | BCL2 |  |  |  |  |  |

Table S3 The core-subnetwork analysis of genes enriched in PI3K-Akt signaling pathway by CytoNca

| Rank | node_name | Degree | Eigenvector | LAC | Betweenness | Closeness | Network |
| --- | --- | --- | --- | --- | --- | --- | --- |
| 1 | MTOR* | 76 | 0.21 | 46.74 | 50.63 | 0.90 | 70.16 |
| 2 | PIK3CA* | 76 | 0.21 | 46.00 | 50.68 | 0.90 | 69.38 |
| 3 | TP53* | 74 | 0.20 | 46.70 | 44.83 | 0.88 | 67.95 |
| 4 | VEGFA* | 72 | 0.20 | 45.56 | 43.68 | 0.86 | 64.51 |
| 5 | PIK3R1* | 72 | 0.20 | 46.00 | 38.49 | 0.86 | 64.65 |
| 6 | EGFR* | 72 | 0.20 | 47.11 | 37.44 | 0.86 | 65.79 |
| 7 | ERBB2* | 72 | 0.20 | 47.56 | 35.50 | 0.86 | 66.18 |
| 8 | AKT1* | 72 | 0.19 | 43.00 | 82.11 | 0.86 | 62.18 |
| 9 | IL6* | 68 | 0.18 | 40.35 | 40.49 | 0.81 | 55.64 |
| 10 | CCND1* | 68 | 0.19 | 44.71 | 32.11 | 0.81 | 59.61 |
| 11 | HSP90AA1* | 66 | 0.19 | 44.85 | 26.74 | 0.80 | 57.93 |
| 12 | MAPK1* | 64 | 0.18 | 43.38 | 28.23 | 0.80 | 54.54 |
| 13 | JAK2* | 62 | 0.18 | 44.26 | 17.95 | 0.78 | 52.86 |
| 14 | MAPK3* | 62 | 0.18 | 41.94 | 26.88 | 0.78 | 51.78 |
| 15 | MCL1* | 58 | 0.16 | 39.17 | 43.98 | 0.74 | 47.30 |
| 16 | JAK1* | 56 | 0.17 | 41.29 | 14.87 | 0.73 | 46.85 |
| 17 | MDM2* | 56 | 0.17 | 42.14 | 11.72 | 0.73 | 47.53 |
| 18 | IL2* | 54 | 0.16 | 40.59 | 10.93 | 0.72 | 45.02 |
| 19 | KDR | 52 | 0.16 | 40.62 | 8.39 | 0.72 | 44.07 |
| 20 | KIT | 50 | 0.15 | 36.16 | 12.51 | 0.70 | 39.14 |
| 21 | MET | 48 | 0.15 | 38.33 | 5.35 | 0.68 | 39.79 |
| 22 | PTK2 | 48 | 0.15 | 36.50 | 8.89 | 0.69 | 38.88 |
| 23 | IGF1R | 48 | 0.15 | 37.83 | 7.27 | 0.69 | 39.69 |
| 24 | FGFR1 | 46 | 0.14 | 36.17 | 5.54 | 0.67 | 38.05 |
| 25 | PIK3CD | 46 | 0.13 | 30.61 | 11.97 | 0.68 | 32.67 |
| 26 | PIK3CG | 46 | 0.13 | 30.78 | 91.65 | 0.68 | 32.84 |
| 27 | MAP2K1 | 46 | 0.15 | 38.78 | 4.68 | 0.68 | 40.48 |
| 28 | PDGFRB | 46 | 0.14 | 35.83 | 6.87 | 0.68 | 36.93 |
| 29 | NTRK1 | 44 | 0.13 | 32.55 | 9.89 | 0.66 | 35.58 |
| 30 | JAK3 | 44 | 0.13 | 32.00 | 9.45 | 0.66 | 34.41 |
| 31 | FLT3 | 38 | 0.12 | 28.00 | 5.91 | 0.63 | 29.22 |
| 32 | NFKB1 | 38 | 0.11 | 28.63 | 6.88 | 0.64 | 30.27 |
| 33 | PGF | 36 | 0.10 | 24.67 | 7.08 | 0.62 | 26.39 |
| 34 | SYK | 34 | 0.10 | 25.65 | 4.13 | 0.62 | 26.42 |
| 35 | FLT1 | 34 | 0.11 | 27.29 | 2.92 | 0.61 | 28.12 |
| 36 | CDK4 | 34 | 0.11 | 29.18 | 1.41 | 0.61 | 30.24 |
| 37 | INSR | 32 | 0.10 | 23.50 | 14.95 | 0.61 | 24.93 |
| 38 | CDK2 | 30 | 0.09 | 24.80 | 2.32 | 0.60 | 26.16 |
| 39 | PRKCA | 28 | 0.09 | 22.00 | 1.90 | 0.58 | 22.90 |
| 40 | IKBKB | 26 | 0.08 | 20.00 | 1.94 | 0.58 | 20.80 |
| 41 | BCL2 | 20 | 0.06 | 16.80 | 0.45 | 0.55 | 17.68 |
| 42 | NTRK2 | 18 | 0.06 | 14.67 | 0.24 | 0.54 | 15.53 |
| 43 | GYS1 | 6 | 0.02 | 2.67 | 0.15 | 0.49 | 3.20 |
| 44 | CHRM2 | 2 | 0.00 | 0.00 | 0.00 | 0.41 | 0.00 |

*Genes with scores higher than the median value.

Table S4 Ranking results of 18 target genes by CytoHubba

| node_name | MCC | DMNC | MNC | Degree | EPC | BN | EC | Closeness | Radiality | Betweenness | Stress | CC |
| --- | --- | --- | --- | --- | --- | --- | --- | --- | --- | --- | --- | --- |
| IL2 | 2789705318400 | 2.15337 | 17 | 34 | 6.687 | 1 | 1 | 17 | 2.17647 | 0.39167 | 24 | 0.47415 |
| MTOR | 2789705318400 | 2.15337 | 17 | 34 | 6.682 | 1 | 1 | 17 | 2.17647 | 0.39167 | 24 | 0.47415 |
| EGFR | 2789705318400 | 2.15337 | 17 | 34 | 6.682 | 1 | 1 | 17 | 2.17647 | 0.39167 | 24 | 0.47415 |
| JAK1 | 2789705318400 | 2.15337 | 17 | 34 | 6.672 | 1 | 1 | 17 | 2.17647 | 0.39167 | 24 | 0.47415 |
| MAPK1 | 2789705318400 | 2.15337 | 17 | 34 | 6.641 | 1 | 1 | 17 | 2.17647 | 0.39167 | 24 | 0.47415 |
| CCND1 | 2789705318400 | 2.15337 | 17 | 34 | 6.629 | 1 | 1 | 17 | 2.17647 | 0.39167 | 24 | 0.47415 |
| MCL1 | 2789705318400 | 2.15337 | 17 | 34 | 6.574 | 1 | 1 | 17 | 2.17647 | 0.39167 | 24 | 0.47415 |
| MAPK3 | 2789705318400 | 2.15337 | 17 | 34 | 6.57 | 1 | 1 | 17 | 2.17647 | 0.39167 | 24 | 0.47415 |
| ERBB2 | 2789705318400 | 2.15337 | 17 | 34 | 6.55 | 1 | 1 | 17 | 2.17647 | 0.39167 | 24 | 0.47415 |
| HSP90AA1 | 2789705318400 | 2.15337 | 17 | 34 | 6.525 | 1 | 1 | 17 | 2.17647 | 0.39167 | 24 | 0.47415 |
| MDM2 | 2789705318400 | 2.15337 | 17 | 34 | 6.511 | 1 | 1 | 17 | 2.17647 | 0.39167 | 24 | 0.47415 |
| AKT1 | 1394852659200 | 2.11791 | 16 | 32 | 6.423 | 1 | 0.5 | 16.5 | 2.11765 | 0.26667 | 16 | 0.47581 |
| VEGFA | 2789705318400 | 2.15337 | 17 | 34 | 6.421 | 1 | 1 | 17 | 2.17647 | 0.39167 | 24 | 0.47415 |
| JAK2 | 1394852659200 | 2.11791 | 16 | 32 | 6.395 | 1 | 0.5 | 16.5 | 2.11765 | 0.26667 | 16 | 0.47581 |
| PIK3R1 | 2615348736000 | 2.13586 | 16 | 32 | 6.386 | 1 | 0.5 | 16.5 | 2.11765 | 0.125 | 8 | 0.47984 |
| TP53 | 2789705318400 | 2.15337 | 17 | 34 | 6.322 | 1 | 1 | 17 | 2.17647 | 0.39167 | 24 | 0.47415 |
| IL6 | 174356582400 | 2.08309 | 15 | 30 | 6.209 | 1 | 0.5 | 16 | 2.05882 | 0.125 | 8 | 0.47816 |
| PIK3CA | 2615348736000 | 2.13586 | 16 | 32 | 6.147 | 1 | 0.5 | 16.5 | 2.11765 | 0.125 | 8 | 0.47984 |

BN, BottleNeck; EC, EcCentricity; CC, ClusteringCoefficient.
